# PP68 Economic Burden Of Low Back Pain Treated In Outpatient Care In Brazil From 2010 To 2019

**DOI:** 10.1017/S0266462325102869

**Published:** 2025-12-29

**Authors:** Yara Marques, Maria Augusta Mota, Tais Lacerda, Aline Toledo, Rodrigo Carregaro

## Abstract

**Introduction:**

The healthcare and rehabilitation processes for individuals with low back pain impose significant costs on healthcare systems worldwide. Understanding the economic burden of this condition can help policymakers implement preventive strategies and promote a more rational allocation of health resources. The aim of this study was to investigate the costs of managing low back pain in Brazil between 2010 and 2019.

**Methods:**

This study evaluated the costs of low back pain from the perspective of the Brazilian Unified Health System (SUS) in the outpatient setting. Data were collected from the Ambulatory Information System and analyzed by sex, age group, and health condition (low back pain). Ambulatory care data were presented as number of visits, total annual cost, and number of procedures performed. In addition, linear regression analysis using generalized linear models (gamma distribution with identity link) was performed to examine the association between total outpatient care costs and predictors such as age, gender, and race.

**Results:**

Between 2010 and 2019, the SUS spent more than USD189 million to treat low back pain in adults. During this period, more than 16 million physical therapy sessions, 7,000 surgeries, and one million imaging studies were performed. These procedures accounted for approximately 85 percent of total spending on low back pain in Brazil. Despite the high number of physical therapy sessions, significant expenditures were still observed for surgical procedures. Regression analysis showed that men had higher costs per procedure than women.

**Conclusions:**

The study revealed significant costs for low back pain in Brazil, with higher expenditures for men and those aged 34 to 63 years. Major expenditures were associated with surgery and physical therapy, whereas the prescription of imaging studies decreased, which is in line with recommendations. These findings highlight the need for targeted strategies to effectively manage resources for low back pain in the ambulatory care setting.

